# Poor Efficacy of L-Acetylcarnitine in the Treatment of Asthenozoospermia in Patients with Type 1 Diabetes

**DOI:** 10.3390/jcm8050585

**Published:** 2019-04-28

**Authors:** Rosita A. Condorelli, Aldo E. Calogero, Rossella Cannarella, Filippo Giacone, Laura M. Mongioi’, Laura Cimino, Antonio Aversa, Sandro La Vignera

**Affiliations:** 1 Department of Clinical and Experimental Medicine, University of Catania, 95123 Catania, Italy; rosita.condorelli@unict.it (R.A.C.); acaloger@unict.it (A.E.C.); roxcannarella@gmail.com (R.C.); filippogiacone@yahoo.it (F.G.); lauramongioi@hotmail.it (L.M.M.); lauracimino@hotmail.it (L.C.); 2 Department of Experimental and Clinical Medicine, Magna Graecia University of Catanzaro, 88100 Catanzaro, Italy; aversa@unicz.it

**Keywords:** type 1 diabetes, asthenozoospermia, carnitine

## Abstract

**Introduction**. In recent years, research has focused on the impact that diabetes mellitus (DM) has on male reproductive function. The available evidence has mainly considered type 2 DM (DM2). However, we have previously shown that type 1 DM (DM1) also affects male reproductive health. Given the efficacy of carnitine in the treatment of male infertility, a topic that merits further investigation is its role in the treatment of infertile patients with DM1. **Aim.** To investigate the efficacy of carnitines for the treatment of asthenozoospermia in DM1 patients. **Methods.** This was a two-arm single-blind, randomized control trial. The patients enrolled in this study were assigned to the group receiving L-acetylcarnitine (LAC) (1.5 g daily for 4 months) or to the group receiving LAC (same dosage) plus L-carnitine (LC) (2 g daily for 4 months). Serum-glycated hemoglobin levels did not differ significantly after either of the two treatments given. Administration of LAC plus LC showed greater efficacy on progressive sperm motility than single therapy (increase 14% vs. 1% after treatment, respectively). **Discussion.** The results of this study showed that the administration of LAC plus LC is more effective than the administration of LAC alone. The lower efficacy of LAC alone could be due to the lower overall administered dosage. Alternatively, a selective defect of carnitine transporters at an epididymal level could be hypothesized in patients with DM1. Further studies are needed to clarify this point.

## 1. Introduction

In recent years, scientific interest for the potential male reproductive consequences of diabetes mellitus (DM) has been growing [1]. The greatest attention has been given to patients with type 2 DM (DM2), mainly because of its greater prevalence and clearer pathogenic mechanisms (hypogonadism, oxidative stress, neuropathy, sexual dysfunction) [1,2,3,4]. However, data concerning sperm quality also emerge in type 1 DM (DM1) patients. The latter is a disease with a different pathophysiology compared with DM2, being characterized by an absolute lack of insulin secretion compared to the condition of insulin-resistance typically found in DM2 patients [5,6]. In particular, a recent meta-analysis has shown a significant decrease in the volume of ejaculate in DM1 patients [7].

We previously reported a significant decrease in the sperm motility of DM1 patients, suggesting the need to evaluate a complication that is not usually considered. In our previous study, we showed that specific alteration of the sperm mitochondrial membrane potential in these patients occurs some years before sperm motility decline becomes evident during sperm analysis [5,6].

The pharmacological treatment of asthenozoospermia is very controversial, and hormonal and non-hormonal therapeutic options may be used. L-acetylcarnitine (LAC) and/or L-carnitine (LC) are often used in these patients. The rationale for its use is based on the potential antioxidant and prokinetic effects. Several lines of clinical evidence have been published showing different treatment protocols using single or combined strategies [8]. A recent meta-analysis of the literature has shown that LC improves sperm parameters, placing it among the treatment options for idiopathic infertility together with other possible treatments, such as pentoxifylline, coenzyme Q10, follicle-stimulating hormone, tamoxifen, and kallikrein [9].

Total and free carnitine levels have been reported to be low in patients with DM1. This decrease is related to the duration of the disease, and could potentially affect long-term complications in these patients [10,11,12].

The potential causes of the carnitine decrement found in DM1 patients are many. It may be due to lower dietary intake, decreased intestinal absorption, and/or increased renal excretion. At the same time, the chronic consequences associated with this deficiency are not completely known. We have no evidence regarding the consequences of this deficiency on sperm parameters or the effects of integration in DM1 patients [10,11,12].

Therefore, the present study was undertaken to evaluate the effects on sperm parameters of the administration of LAC alone or in combination with LC in DM1 patients with asthenozoospermia.

## 2. Patients and Methods

The study was conducted on 40 asthenozoospermic patients with DM1 aged 18–35 years. Patients with any of the following conditions were excluded from the study: chronic complications of diabetes (neuropathy, nephropathy, retinopathy); dyslipidemia; hypertension; body mass index ≥ 30.0 kg/m^2^; presence of mono- and/or bilateral varicocele; diagnosis of male accessory gland infection/inflammation; congenital and/or acquired obstruction of proximal and/or distal seminal ducts; testicular volume <12 mL (according to Prader’s orchidometer); hypogonadism, defined by total testosterone <3 ng/mL or 10.4 nM; hyperprolactinemia, defined by a level above the upper limit of normal after a single measurement of serum levels; hyperestrogenism; thyroid disorders; cryptorchidism; cigarette smoking; recent drug and/or alcohol abuse. All patients had a basal bolus scheme of insulin therapy with an exogenous insulin/body weight ratio of 0.5/kg.

Two semen samples, 2–3 weeks apart, were obtained from each patient before and after 4 months of pharmacological treatment. The mean values of both measurements were considered for the statistical analysis.

A blood sample was collected from the antecubital vein, at 08:30, to measure serum concentrations of luteinizing hormone, follicle-stimulating hormone, total testosterone, 17β-estradiol, prolactin, and glycated hemoglobin (HbA1c). HbA1C was re-evaluated after 4 months for clinical control assessment.

All patients provided written informed consent before enrollment, and were aware that their data would be used for clinical research purposes. The protocol was approved by the internal review board of Operative Unit of Andrology and Endocrinology Policlinico G. Rodolico University Hospital, Catania, Italy. (approval no. 07/2018).

Patients were divided into two groups for randomly received treatment: Group A was given LAC 500 mg tablets every 8 h every day for 4 months, and group B received the same dose of LAC plus LC 2 g oral solution once daily for 4 months. Randomization was based on a single sequence of random assignments generated from PC (simple randomization) in a single-blind procedure.

## 3. Sperm Analysis

Semen samples were collected by masturbation into a sterile container after 3–5 days of sexual abstinence. After liquefaction, semen was analyzed according to the World Health Organization criteria (WHO, 2010). Semen analysis was performed by the same technician (FG).

## 4. Measurement of Serum Hormone Concentrations

The hormone assays were performed by electrochemiluminescence with a Roche/Hitachi device (Cobas 6000; Roche Diagnostics, Indianapolis, IN, USA). The reference intervals were as follows: Luteinizing hormone 1.6–9.0 mUI mL^−1^, Follicle Stimulating Hormone = 2.0–12.0 mUI mL^−1^, 17β-estradiol = 8.0–43.0 pg mL^−1^, total testosterone = 2.8–8.0 ng mL^−1^, prolactin = 4.0–15.0 ng mL^−1^.

## 5. Statistical Analysis 

All statistical analyses were performed using SPSS v. 19 software (SPSS Inc, IBM Corp, Somers, NY, USA). Continuous variables, presented as median (interquartile range), were tested by Mann–Whitney *U* test or Kruskal–Wallis test according to their non-normal distribution (normality of variable distribution was tested by Kolmogorov–Smirnov test). A *p*-value lower than 0.05 was considered statistically significant. Spearman’s or Pearson’s correlation coefficients were used to test the associations between different variables.

## 6. Results

**Figure 1:**
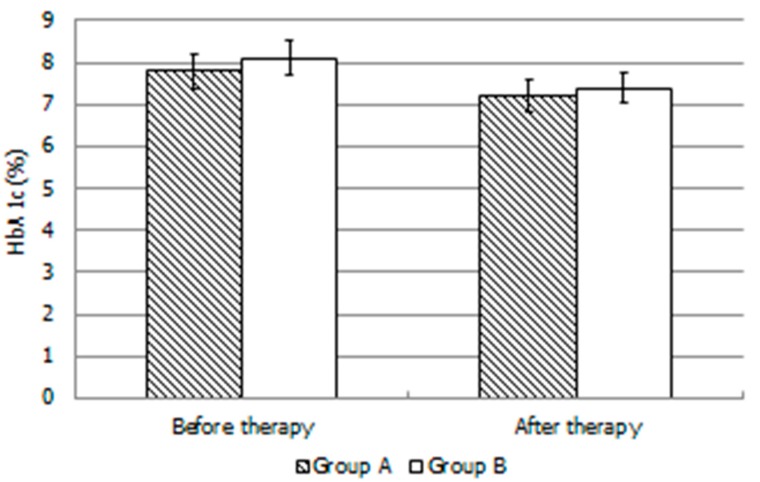
Glycated hemoglobin levels in each group of patients with type 1 diabetes mellitus before and after administration of L-Acetyl-Carnitine or L-Acetyl-Carnitine plus L-Carnitine. Type 1 diabetes mellitus (DM1) (a) L-acetylcarnitine (LAC) 500 mg tablets every 8 h every day for 4 months; DM (b) LAC 500 mg tablets every 8 h and LC 2 g oral solution only once a day for 4 months. Values are shown as mean ± SD.

There were no drop-out patients as all completed the study protocol. Following randomization, group A and B did not differ significantly for the following parameters: age (25.0 ± 2.0 vs. 27.0 ± 2.0 years), body mass index (22.0 ± 1.0 vs. 23.0 ± 1.0 kg/m^2^), disease duration (12.0 ± 2.0 vs. 14.0 ± 3.0 years), HbA1c (7.8 ± 1.2 vs. 8.1 ± 1.5 %). HbA1c values were also similar after 4 months of treatment (7.2 ± 1.3 vs. 7.4 ± 1.3 %) (Figure 1). Compared to at enrollment, HbA1c values decreased slightly in both groups, but the difference was not statistically significant. HbA1c levels did not correlate with any of the evaluated sperm parameters.

**Figure 2:**
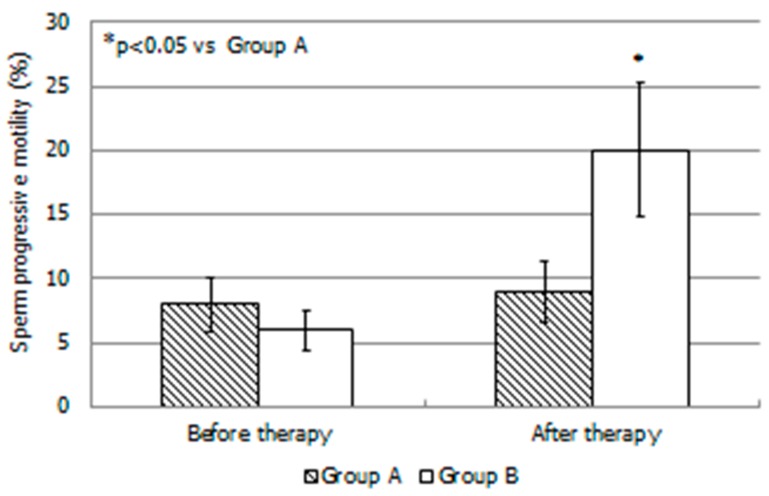
Sperm progressive motility in each group of patients with type 1 diabetes mellitus before and after administration of L-Acetyl-Carnitine or L-Acetyl-Carnitine plus L-Carnitine. DM1 (a) LAC 500 mg tablets every 8 h every day for 4 months; DM (b) LAC 500 mg tablets every 8 h and LC 2 g oral solution only once a day for 4 months. Values are shown as mean ± SD; * *p* < 0.05 compared to DM1 (a).

At the time of enrollment, groups A and B did not differ in anthropometric, seminal, and hormonal parameters (Table 1). After 4 months of treatment, DM1 patients treated with LAC plus LC showed significantly higher progressive sperm motility compared with DM1 patients treated with LAC alone (Table 2 and Figure 2). In group A, after treatment, we observed a significant increase (*p* < 0.05) of progressive sperm motility in two patients (10%). In group B, 10 patients (50%) had a significant improvement (*p* < 0.05) of this parameter compared to baseline. No patient of the two groups showed a progressive motility value of >32% after therapy, a value considered to be the lower threshold for this parameter (WHO, 2010).

## 7. Discussion

The results of the present study showed that the administration of LAC (1.5 g daily for 4 months)—a treatment currently adopted in the andrological clinical practice to improve sperm motility [8] is not effective for the treatment of asthenozoospermia in DM1 patients. By contrast, the concomitant administration of LAC plus LC (2 g daily) to age-matched DM1 patients was shown to be efficacious in improving progressive sperm motility. After the 4-month-long administration, both groups showed a slight but no significant decrease in HbA1c levels.

Carnitine is a short-chain non-protein amino acid. It is a carrier of fatty acids, allowing mitochondria to use them for the production of Adenosin triphospfate. The fatty acids must be activated within the cytosol before being degraded. Their complete degradation then occurs in the mitochondria instead. The activated fatty acids are found in the form of acyl-CoA, a fatty acid linked to a molecule of coenzyme A. However, acyl-CoA is not able to cross the mitochondrial membrane due to the presence of its acyl portion. The acyl portion is then transferred to a carnitine molecule, forming acylcarnitine [8].

Carnitines display its positive effects on human general health, particularly in LC formulation, which is derived from endogenous synthesis and food intake. LC acts as a transporter for long-chain fatty acids into the mitochondria, in turn facilitating energy and LAC production. The latter is synthetized by the L-carnitine acetyltransferase, which is responsible for mitochondrial coenzyme A (CoA) and acyl-CoA concentrations [13,14,15].

The presence of a relationship between carnitine and DM has been established. Subjects of more debate are concerned with lower carnitine serum levels, their possible predictive role in the diagnosis of DM1, and efficacy in the treatment of diabetic neuropathy. In particular, several studies have highlighted decreased carnitine levels in DM1 and DM2 patients, and the reasons for this are unclear. Possible explanations may be lower absorption capacity, an unbalanced diet, or increased excretion through urine [12]. Recently, a meta-analysis carried out on four different global studies, including 284 patients, evaluated the efficacy of LC administration in DM2 patients, reporting an improvement of glycemia, total cholesterol, low-density-lipoprotein (LDL) cholesterol, B100, and A1 apolipoproteins, with a 2–3 daily administration [16]. Carnitine deficiency can hinder the entry of fatty acids into the mitochondrial membrane, leading to weight increase, low muscular tone, increased cytosolic concentration of triglycerides, excessive appetite, and glycemic fluctuations [16].

Concerning DM1, the results are promising despite the low quantities of evidence. Accordingly, carnitine deficiency in the first few days of neonatal life has been addressed as a possible cause of the development of DM1 [11]. Indeed, molecules belonging to the carnitine family remove autoreactive immune cells responsible for the disease. In summary, low carnitine levels during neonatal life may cause predisposition to DM1 [11].

Mamoulakis and colleagues (2004) highlighted the decreased total and free carnitine levels in 47 DM1 patients compared to controls, and showed a relationship with disease duration, suggesting a correlation with the complications of the disease [12].

Several lines of evidence have highlighted the neurotrophic and analgesic effects of LAC in the treatment of diabetic neuropathy. Hence, LAC is currently prescribed in the clinical practice for the treatment of this complication [17].

No data are available concerning the possible beneficial effects of carnitine in DM patients with sperm parameter abnormalities. On the contrary, a number of studies confirm carnitine pro-kinetic and antioxidant activity in male idiopathic infertility [8]. A recent meta-analysis confirms this effect while underlining two aspects: (a) limited evidence regarding pregnancy and live birth rate; (b) the controversial methodological aspects of the analyzed studies (Omar et al., 2019). Overall, the quality of the studies and the efficacy of carnitine for the treatment of idiopathic male infertility should be considered moderate.

Moncada and colleagues reported a significant improvement of sperm motility in 20 patients with idiopathic oligoasthenoteratozoospermia after the 60-day-long administration of LAC at a daily dose of 4 g [18]. In the studies by Costa et al. [19] and Vitali et al. [20] the 3 g daily administration of LC for 3–4 months was effective in ameliorating sperm motility [19,20]. Subsequently, several studies have been carried out using a combination of LC and LAC at a daily dosage of 2 and 1 g, respectively, for 3 months, or at the same dosage for six months. Finally, Balercia and colleagues (2005) adopted a therapeutic scheme of 3 g daily administration of LAC and LC for 6 months [21].

The interpretation of the results of the present study is not unique. High carnitine concentrations occur at the epididymal level. LC is actively transported into the epididymis through specific transporters known as carnitine/organic cation transporters (OCTNs). The OCTN isoform 1 (OCTN1) shows a low specificity for carnitine. On the contrary, OCTN2 has a high specificity for carnitine at the epididymal level [13,14,15]. No data have currently been reported on the expression of OCTNs in epididymal spermatozoa in DM, neither in animal models nor in humans. This topic deserves further investigation. It will be necessary to evaluate the existence of a selective deficit of this category to understand the differences in the efficacy of LC plus LAC compared to LAC alone on progressive sperm motility in DM1 patients.

At the same time, the LC and LAC dosage adopted in this study as well as the treatment duration may be considered insufficient when compared with previous reports of the literature [8].

Finally, further studies should be performed to investigate mitochondrial function in DM patients before starting the treatment to assess whether a low mitochondrial membrane potential may influence the effectiveness of carnitine and, in turn, the dosage to be prescribed. We have previously shown that an alteration of this parameter promotes asthenozoospermia in DM1 patients [6].

Similar considerations should also be applied to the epididymal US feature, since signs of inflammation or neuropathy on US have been revealed to promote the diagnosis [3,5].

## 8. Study Limitations

The main limitations of this study are the low sample size, the unavailability of biofunctional sperm parameters (mitochondrial function and α1-glycosidases levels, the latter being a marker of epididymal secretory function) as well as of the epididymal ultrasound characteristics. Another limitation concerns the lack of a true control arm (placebo) and, also, the possibility that more frequent sampling in a more extended protocol might be of interest. According to the current guidelines [22], DM1 patients are not considered at risk for infertility. Therefore, information derived from their sperm quality is important for a better understanding of biochemical mechanisms and possible future clinical applications.

## Figures and Tables

**Table 1:** Anthropometric, seminal, and hormonal characteristics of patients at enrollment.

Parameters	Group A (*n* = 20)	Group B (*n* = 20)	*p*-value
**Anthropometric parameters**			
Age (years), median (IQR)	25.0 (22.0–33.0)	27.0 (24.0–35.0)	0.30
Weight (kg), median (IQR)	69.0 (64.5–76.5)	71.0 (60.5–77.0)	0.69
Height (m), median (IQR)	176.0 (170.0–179.0)	174.5 (168.5–178.0)	0.30
Body mass index (kg/m^2^), median (IQR)	23.0 (21.25–24.75)	24.0 (22.25–29.75)	0.30
Waist circumference (cm), median (IQR)	86.0 (81.25–88.0)	88.0 (82.0–90.0)	0.25
**Sperm parameters**			
Volume (mL), median (IQR)	1.8 (1.6–3.0)	1.9 (1.6–3.2)	0.97
Concentration (mil/mL), median (IQR)	25.0 (20.0–45.0)	28.0 (22.0–50.0)	0.26
Progressive motility (%), median (IQR)	8.0 (2.0–12.0)	7.0 (3.0–15.0)	0.69
Normal forms (%), median (IQR)	8.0 (6.0–14.0)	7.0 (5.0–15.0)	0.72
Leucocytes (mil/mL), median (IQR)	0.4 (0.30–0.80)	0.5 (0.2–0.8)	0.96
**Hormonal parameters**			
LH (UI/L), median (IQR)	2.3 (1.72–2.57)	2.1 (0.8–2.55)	0.22
FSH (UI/L), median (IQR)	2.9 (2.50–3.50)	2.6 (0.32–3.3)	0.30
TT (ng/mL), median (IQR)	6.2 (5.0–8.2)	6.4 (4.8–8.2)	0.30
E2 (pg/mL), median (IQR)	9.0 (6.25–11.5)	8.6 (5.0–13.75)	0.24
PRL (ng/mL), median (IQR)	10.5 (7.25–12.75)	12.0 (6.0–16.0)	0.26

**Legend:** DM1 (a) LAC 500 mg tablets every 8 h every day for 4 months; DM (b) LAC 500 mg tablets every 8 h and LC 2 gr oral solution only once a day for 4 months.

**Table 2:** Sperm parameters before and after 4 months of pharmacological treatment.

Parameter	Group Aat Enrollment	Group Aafter Treatment	Group Bat Enrollment	Group Bafter Treatment
Volume (mL), median (IQR)	1.8 (1.6–3.0)	2.0 (1.5–3.2)	1.9 (1.6–3.2)	2.3 (1.5–3.5)
Concentration (mil/mL), median (IQR)	25.0 (20.0–45.0)	29.0 (22.0–49.0)	28.0 (22.0–50.0)	32.0 (24.0–51.0)
Progressive motility (%), median (IQR)	8.0 (2.0–12.00)	9.0 (2.0–14.00)	7.0 (3.0–15.00)	20.0 * (5.0–28.00)
Normal forms (%), median (IQR)	8.0 (6.0–14.0)	10.0 (7.0–16.0)	7.0 (5.0–14.0)	11.0 (7.0–18.0)
Leucocytes (mil/mL), median (IQR)	0.4 (0.3–0.8)	0.5 (0.3–0.8)	0.5 (0.2–0.8)	0.3 (0.1–0.6)

**Legend:** Values of two seminal evaluations before and after pharmacological treatment. DM1 (a) LAC 500 mg tablets every 8 h every day for 4 months. DM (b) LAC 500 mg tablets every 8 h and LC 2 g oral solution only once a day for 4 months. * *p* < 0.05 compared to DM1 (a).
